# Association of Symptoms of Gastroesophageal Reflux with Metabolic Syndrome Parameters in Patients with Endocrine Disease

**DOI:** 10.1155/2014/863206

**Published:** 2014-01-30

**Authors:** Masatoshi Nomura, Naotaka Tashiro, Tetsuhiro Watanabe, Akie Hirata, Ichiro Abe, Taijiro Okabe, Ryoichi Takayanagi

**Affiliations:** ^1^Department of Medicine and Bioregulatory Science, Graduate School of Medical Sciences, Kyushu University, 3-1-1 Maidashi, Higashi-ku, Fukuoka 812-8582, Japan; ^2^Department of Preventive Medicine, Graduate School of Medical Sciences, Kyushu University, 3-1-1 Maidashi, Higashi-ku, Fukuoka 812-8582, Japan

## Abstract

*Background*. Metabolic syndrome (MetS) and obesity are known risk factors for gastroesophageal reflux disease (GERD), which is often found in patients with endocrine disorders, such as thyroid dysfunction and hypopituitarism. To clarify the relationship of endocrine disease with GERD, we investigated the symptoms of GERD in patients with various endocrine diseases. *Methods*. Patients with various endocrine disorders who visited Kyushu University Hospital were included. GERD symptoms were examined using a self-administered questionnaire, the frequency scale for the symptoms of GERD (FSSG). Metabolic parameters, including body-mass index (BMI), waist circumference, blood pressure, hemoglobin A1c, total cholesterol, high-density lipoprotein cholesterol (HDL-C), and triglycerides, and values of endocrine function, including thyroid stimulating hormone, free thyroxin, cortisol, and insulin-like growth factor-1, were assessed. *Results*. A total of 111 consecutive patients were recruited for the study. Among these, 18 (16.2%) patients were considered to have GERD. Among the parameters, BMI (*P* = 0.03) and triglycerides (*P* = 0.001) showed a positive association and HDL-C (*P* = 0.0007) showed an inverse association with the FSSG score. However, none of the endocrine values were associated with the FSSG score. *Conclusion*. Symptoms of GERD in patients with endocrine disorders might be attributed to MetS as comorbidity.

## 1. Introduction

Metabolic syndrome (MetS) is a cluster of metabolic abnormalities defined as the presence of three or more of the following factors: abdominal obesity (increased waist circumference), elevated triglycerides, low high-density lipoprotein cholesterol (HDL-C) levels, high blood pressure, and high fasting plasma glucose levels [1]. MetS is a high risk factor for cardiovascular and other atherosclerotic diseases [2]. Obesity has been implicated in various gastrointestinal diseases, such as gastroesophageal reflux disease (GERD). The prevalence of GERD has been increasing worldwide [3], and adversely affects health-related quality of life [4]. Dyspepsia is usually defined as upper abdominal pain or retrosternal pain, discomfort, belching, abdominal bloating, nausea, or other symptoms considered to have arisen from the upper alimentary tract. Reflux symptoms (RS), such as heartburn and regurgitation, are regarded as typical symptoms of GERD. However, it is reported that other dyspeptic symptoms are also common in patients with nonerosive GERD [5, 6]. Therefore, GERD is also associated with dyspeptic manifestations other than RS. Dyspeptic symptoms that respond to proton pump inhibitors are classified as acid-related dyspepsia (ARD) [7]. Because a complex of RS with or without mucosal damage and/or complications are observed in GERD [8], its diagnosis requires accurate determination of symptoms. The majority of patients with typical RS have no evidence of erosive esophagitis [9], and nonerosive GERD is a difficult disorder to diagnose because even typical RS depend on the patient's description and are difficult to define in certain populations. To overcome these difficulties, Kusano et al. developed a questionnaire, the Frequency Scale for the Symptoms of GERD (FSSG) [10]. The FSSG contains 12 symptoms most commonly experienced by GERD patients, with seven of the 12 being RS and the remaining five ARD. There is a significant positive correlation between RS and ARD. GERD patients suffer not only from RS, but also from ARD [11]. FSSG has been estimated to be clinically useful for the initial diagnosis of GERD. Patients with FSSG scores of more than 8 were considered as positive.

Endocrine disorders are common, and the effects of endocrine disorders present with a wide range of clinical manifestations. Hormonal interactions among the systems throughout the body are not fully understood. Many vague clinical symptoms may in fact be manifestations of underlying endocrine disease. In fact, the gastrointestinal manifestations of common endocrine diseases may aid in the diagnosis of neuroendocrine abnormalities. For example, chronic dyspeptic symptoms, such as epigastric pain and fullness, as well as eructation, nausea, and vomiting, are frequently observed in patients with thyroid dysfunction [12]. Digestive symptoms or signs may also reveal signs of thyroid disease and, when ignored or underestimated, diagnosis may be delayed and serious consequences may occur [13]. In addition, patients with adult GH deficiency (GHD) present with features, such as abdominal obesity, dyslipidemia, and insulin resistance with MetS [14]. Therefore, GHD may cause gastrointestinal manifestations, such as GERD.

In this study, we investigated the link between endocrine parameters and GERD. To evaluate the prevalence of GERD in patients with various endocrine disorders, including MetS, we investigated the frequency of GERD as a complication in patients with endocrine diseases by using the FSSG.

## 2. Methods

### 2.1. Study Subjects

The subjects of this study were 127 consecutive patients who visited Kyushu University Hospital between April 2009 and March 2010 with various endocrine disorders. All patients had appropriate treatment. Thirty-five male and 92 female patients were enrolled. After 16 patients taking antipeptic drugs, proton pomp inhibitors, or histamine H2 blockers were excluded, 111 participants remained in the study. Diagnoses of these patients are shown in Table 2. Informed consent was obtained before the start of the study in conformity with the Declaration of Helsinki, and the study was approved by the institutional review board of Kyushu University Hospital.

### 2.2. Questionnaire

The FSSG questionnaire was completed by the patients. As previously reported [11], the 12 questions were divided into those covering RS and ARD symptoms, and a score for each category was examined. There is a maximum score of 48 points for the questionnaire, comprising up to 28 points for RS and 20 points for ARD symptoms. There was a significant positive correlation between RS and ARD (Spearman's rank correlation coefficient = 0.47 (*P* < 0.01)).

### 2.3. Anthropometry

Height, weight, and waist circumference were measured while subjects wore light clothing and no shoes. Body-mass index (BMI) was calculated as weight divided by the square of height (kg/m^2^). Waist circumference was measured at the midpoint between the lower border of the rib cage and the iliac crest. Blood pressure was measured at the end of the physical examination with the patient in a sitting position, and all participants were allowed at least 5 min of rest before the measurements.

### 2.4. Lipid and Endocrine Profiles

All measurements were obtained in the morning after overnight fasting. Total cholesterol (TC), HDL-C, and triglyceride were measured with an enzymatic color test (Daiichi, Hitachi 747, Tokyo). Serum cortisol and insulin-like growth factor-1 (IGF-I) levels were measured by specific radioimmunoassay. Serum free thyroxin (T4) and thyroid stimulating hormone (TSH) levels were measured by ELISA.

### 2.5. Statistical Analysis

To investigate the contribution of metabolic syndrome and endocrine disorders to GERD symptoms, the subjects were divided into two groups: those with an FSSG score ≥ 8 and those with an FSSG score ≤ 7. Between-group comparisons were made by *χ* ^2^ test for gender, *t*-test for age, and Wilcoxon rank sum test for other variables. Because only one person had IGF-1 levels measured in the FSSG ≥ 8 group, the difference of the median of IGF-1 was not tested. Association of clinical factors with the FSSG score was assessed by linear regression analysis, and standardized partial regression coefficients were calculated. Logistic regression analysis was used to estimate odds ratios (ORs) and 95% confidence intervals (95% CIs) of GERD defined by FSSG ≥ 8 for various clinical parameters. Statistical adjustment was made for sex, age (year), and diagnoses of subjects (thyroid disease, adrenal disease, acromegaly, or others) in linear regression analysis. Further BMI was adjusted in logistic regression analysis. Statistical significance was declared if two-sided *P* value was < 0.05. Statistical analyses were performed using SAS version 9.2 (SAS Institute, Cary, NC, USA).

## 3. Results

### 3.1. Characteristics of Subjects Enrolled in the Present Study

The study characteristics are shown in Table 1. A total of 111 consecutive patients (81 women and 30 men; age range, 38–76 years) were analyzed in this study. Since antipeptic treatment has a clear impact on the frequency and intensity of GERD symptoms, patients taking antipeptic drugs, proton pomp inhibitors, or histamine H2 blockers were excluded. Of these, 18 (17.3%) patients were considered to have GERD according to the criteria for the FSSG. The diagnoses of the patients and the number of the patients with FSSG ≥ 8 are summarized in Table 2. The patients were diagnosed with endocrine diseases, such as Cushing's syndrome (*n* = 4), adrenal insufficiency (*n* = 11), Basedow's disease (*n* = 34), hypothyroidism (*n* = 31), acromegaly (*n* = 6), nonfunctioning adrenal tumor (*n* = 4), and simple obesity (*n* = 4). Patients in this study were properly treated as indicated by the interquartile range in Table 1.

### 3.2. Contribution of Metabolic and Endocrine Parameters to GERD Symptoms

Comparisons of each parameter between the groups are shown in Table 3. Triglyceride levels in patients with an FSSG score ≥ 8 were significantly higher than those in patients with an FSSG score ≤ 7 (*P* = 0.04). Diastolic blood pressure was significantly lower in the FSSG score ≥ 8 group than in the FSSG score ≤ 7 group. Unexpectedly, neither free T4 nor TSH was significantly different between the FSSG score groups.

To investigate the relationship between each parameter and FSSG scores, multiple regression analysis was performed.  Table 4 shows the results of the association between FSSG score and various metabolic parameters, serum chemistry, and hormonal values. After adjusting for sex, age and diagnoses of subjects, among metabolic parameters, BMI and waist circumference showed a positive correlation with RS scores but not with ARD scores. However, blood pressure showed no significant association. Among serum chemistry, triglyceride and HDL-C levels showed a positive and inverse association with RS and ARD scores. Notably, all of these parameters, BMI, triglyceride, and HDL-C values, are diagnostic factors for metabolic syndrome. Uric acid showed a positive correlation with RS. Because BMI is strongly and positively related to the frequency of symptoms of GERD [15], the variables were further adjusted for BMI. Triglyceride and HDL-C levels showed a positive and inverse association with the RS and ARD scores. However, uric acid no longer showed a significant association (*P* for RS = 0.27), suggesting that uric acid value is closely associated with BMI. These results are consistent with the previous observation that the prevalence of GERD is higher in subjects with metabolic syndrome [16]. None of the endocrine values, including serum cortisol, free T4, TSH, and IGF-1 levels, showed a significant association with the FSSG score.

The associations of metabolic and endocrine parameters and the risk of the manifestation of GERD symptoms were also analyzed. The OR of the prevalence of GERD, assessed by the FSSG score, in those who had hypertriglycemia was 5.85 compared with that in subjects who had a triglyceride level below 150 mg/dL (Table 5). Low HDL-C levels increased the OR up to 23.06. In contrast, hyperthyroidism (free T4 ≥ 1.27 ng/mL), hypothyroidism (TSH ≥ 1.28 ng/mL), and hypercortisolemia (cortisol ≥ 9.5 *μ*g/dL) did not increase the OR.

## 4. Discussion

In this study, we investigated the association between the FSSG score and endocrine values, as well as MetS parameters in patients with various endocrine disorders. We found that BMI, triglyceride, and HDL-C levels were associated with the FSSG score. Interestingly, all these factors are diagnostic factors for MetS, indicating that the FSSG score may be a reliable factor for the prediction of MetS. The prevalence of MetS is closely related to age and BMI. Therefore, to determine whether there was a significant association between the FSSG score and MetS parameters, the variables were adjusted for BMI in addition to age and sex. Interestingly, triglyceride levels were significantly associated with the FSSG score. An interesting finding of this study was that, among the individual components of MetS, elevated serum triglyceride levels were an independent predictive factor for GERD. Visceral adipose tissue is a precursor to increased lipolysis and free fatty acids, leading to insulin resistance, which is regarded as a primary factor in the mechanisms of MetS [17, 18]. Visceral adipose tissue is also metabolically active and is strongly associated with elevated serum levels of proinflammatory adipokines, including interleukin-6, tumor necrosis factor-*α*, and adiponectin, which may play a role in the development of GERD [19, 20]. Such humoral factors from visceral fat tissue might alter the lower esophageal sphincter pressure or affect esophageal clearance of refluxate [16]. It is interesting to know whether such humoral factors change in the condition of endocrine diseases.

In this study, the patients with various endocrine diseases were enrolled. We therefore adjusted the variables by diagnoses of the subjects to exclude heterogeneity that may affect the results. After adjustment by diagnoses of subjects, we still found that there was no significant association between hormonal values and the FSSG score. These results suggested that endocrine values themselves are not directly associated with GERD symptoms as long as they are within normal limits. The prevalence of MetS in GHD patents is known to be higher than that in the general population [21]. However, replacement therapy of GH does not affect the prevalence of MetS [22]. These results indicate that baseline MetS status and visceral obesity are strong predictors of MetS after GH treatment in GHD patients. Therefore, it is reasonable that IGF-1 levels did not show any association with GERD in this study, although the subject number was limited. Thyroid interactions with the gastrointestinal system have been widely reported [23]. In addition, gastrointestinal motor dysfunction has been widely accepted as the main cause of symptoms; however, many complex phenomena have not yet been completely elucidated [24]. In the present study, the FSSG score showed no association with thyroid parameters, free T4, or TSH. Dysthyroidism, whether in excess or deficiency, has clinical manifestations within different portions of the digestive tract and viscera. Whether these are related to hormone level disturbances alone or are associated with a specific thyroid disease is unknown [25], and the underlying pathophysiology is often complex and has not yet been fully determined. Our results suggest that dysthyroidism may not be associated with GERD symptoms.

A limitation of this study was that participants had not performed upper endoscopy. The recent study has shown that obesity also represents an important risk factor for nonerosive GERD with acid exposure [26]. Although there was a significant correlation between RS and ARD score in this study, in agreement with a previous report [27], a further analysis by an upper endoscopy will be required.

In conclusion, BMI, triglyceride, and HDL-cholesterol are associated with the symptoms of GERD in patients with endocrine disease. Endocrine values, including cortisol, IGF-1, and thyroid hormone, are not associated with the FSSG score. These findings suggest that GERD symptoms in patients with endocrine disorders might be attributed to MetS as comorbidity. Therefore, an intervention of MetS should be considered for the treatment of GERD symptoms in patients with endocrine disorders.

## Figures and Tables

**Table 1:** Characteristics of subjects in the present study.

		Number of subjects
Gender, male/female	30/81	111
Age, years	51.6 ± 15.6	111
BMI, kg/m^2^	23.2 (20.7–25.7)	111
Waist circumference, cm	83.0 (74.0–92.0)	111
SBP, mmHg	120 (110–130)	110
DBP, mmHg	70 (60–80)	110
FBS, mg/dL	100 (88–111)	75
HbA_1_c (NGSP), %	5.6 (5.2–6.2)	43
Triglyceride, mg/dL	98 (74–155)	75
TC, mg/dL	203 (181–229)	75
HDL-C, mg/dL	57 (48–74)	66
Uric acid, mg/dL	5.1 (4.2–6.2)	75
Cortisol, *μ*g/dL	8.9 (6.7–14.2)	31
Free T4, ng/mL	1.26 (1.15–1.62)	48
TSH, ng/mL	1.21 (0.37–2.82)	48
IGF-1, ng/mL	250 (220–260)	5
FSSG score*	2 (0–30)	111
ARD*	2 (0–14)	111
RS*	1 (0–16)	111
7≤/≥8^†^	93/18	111

BMI: body-mass index; SBP: systolic blood pressure; DBP: diastolic blood pressure; TC: total cholesterol; HDL-C: high-density lipoprotein cholesterol. Mean ± standard deviation (SD) or median (interquartile range (IQR)). *Median (range). ^†^Number of subjects.

**Table 2:** Diagnoses of patients in this study.

Disease	Number of subjects	FSSG score ≥ 8
Cushing's syndrome	4	0
Adrenal insufficiency	11	3
Basedow's disease	34	6
Hypothyroidism	31	5
Acromegaly	6	1
Nonfunctioning adrenal tumor	4	0
Simple obesity	4	1
Others	17	2

**Table 3:** Characteristics of subjects stratified by FSSG score.

	FSSG ≤ 7	FSSG ≥ 8	*P* for difference
Number of patients	93	18	
Male (%)	26 (28.0)	4 (22.2)	0.62
Age, years	52.2 ± 14.8	48.2 ± 19.2	0.41
BMI, kg/m^2^	23.1 (20.7–25.2)	24.1 (21.2–26.5)	0.33
Waist circumference, cm	83.0 (74.0–91.0)	79.0 (75.0–93.0)	0.97
SBP, mmHg	120 (110–130)	111 (110–130)	0.33
DBP, mmHg	70 (60–80)	60 (50–70)	0.03
FBS, mg/dL	101 (89–112)	93 (85–107)	0.28
HbA_1_c (NGSP), %	5.6 (5.1–6.2)	5.6 (5.5–6.0)	0.69
Triglyceride, mg/dL	97 (68–144)	153 (87–274)	0.04
TC, mg/dL	203 (182–232)	203 (177–215)	0.57
HDL-C, mg/dL	57 (49–74)	55 (46–65)	0.30
Uric acid, mg/dL	5.1 (4.2–6.2)	4.9 (4.5–6.4)	0.91
Cortisol, *μ*g/dL	9.5 (6.7–14.7)	8.7 (7.0–11.9)	0.62
Free T4, ng/mL	1.26 (1.14–1.64)	1.34 (1.18–1.47)	0.89
TSH, ng/mL	1.21 (0.47–2.72)	1.37 (0.02–3.04)	0.97
IGF-1, ng/mL	240 (148–452)	250*	—

Mean ± standard deviation (SD) or median (interquartile range (IQR)). *P* for difference was calculated by *χ* ^2^ test for gender, *t*-test for age, and Wilcoxon rank sum test for other variables.

*Only 1 person was made an evaluation of IGF-1.

**Table 4:** Association of clinical factors with FSSG score.

	Number	*F* total		*F* ARD		*F* RS	
		Standardized *β*	*P*	Standardized *β*	*P*	Standardized *β*	*P*
BMI	111	0.196	0.05	0.043	0.67	0.311	0.002
Waist circumference	111	0.114	0.25	0.003	0.98	0.215	0.04
SBP	110	0.047	0.67	0.012	0.91	0.073	0.51
DBP	110	−0.094	0.36	−0.064	0.53	−0.105	0.30
FBS	75	−0.072	0.58	−0.151	0.23	0.021	0.88
HbA_1_c (NGSP)	43	−0.035	0.85	−0.131	0.46	0.067	0.72
Triglyceride	75	0.406	0.001	0.292	0.02	0.440	0.0005
TC	75	−0.110	0.39	−0.119	0.34	−0.080	0.54
HDL-C	66	−0.427	0.0009	−0.290	0.01	−0.481	0.0004
Uric acid	75	0.214	0.11	0.090	0.49	0.297	0.03
Cortisol	31	−0.088	0.76	0.095	0.73	−0.253	0.36
Free T4	48	−0.115	0.37	−0.191	0.10	−0.017	0.91
TSH	48	0.014	0.92	0.030	0.80	−0.005	0.92
IGF-1	5	0.248	0.61	−0.186	0.05	0.633	0.51

All variables were adjusted by sex, age, and diagnoses of subjects.

**Table 5:** Clinical parameters and GERD risk.

	Number of subjects (%)		OR (95% CI)*
	FSSG ≤ 7	FSSG ≥ 8	
BMI ≥ 25 kg/m^2^	26/93 (28.0)	7/18 (38.9)	1.54 (0.52–4.57)
Waist circumference^†^	35/93 (37.6)	5/18 (27.8)	0.74 (0.22–2.50)
SBP ≥ 140 mmHg	13/92 (14.1)	2/18 (11.1)	0.75 (0.14–3.97)
DBP ≥ 90 mmHg	3/92 (3.3)	1/18 (5.6)	1.27 (0.10–16.05)
FBS ≥ 110 mg/dL	18/63 (28.6)	2/12 (16.7)	0.49 (0.09–2.63)
Triglyceride ≥ 150 mg/dL	14/63 (22.2)	6/12 (50.0)	5.85 (1.17–29.25)
TC ≥ 220 mg/dL	23/63 (36.5)	2/12 (16.7)	0.33 (0.06–1.76)
HDL-C < 40 mg/dL	1/57 (1.8)	2/9 (22.2)	23.06 (0.88–605.63)
Uric Acid ≥ 7.0 mg/dL	8/63 (12.7)	1/12 (8.3)	0.79 (0.08–8.09)
Cortisol ≥ 9.5 *μ*g/dL	12/23 (52.2)	3/8 (37.5)	0.08 (0.004–1.87)
Free T4 ≥ 1.27 ng/mL	20/40 (50.0)	4/8 (50.0)	1.09 (0.20–5.87)
TSH ≥ 1.28 ng/mL	20/40 (50.0)	4/8 (50.0)	1.04 (0.20–5.42)

*Sex, age, BMI, and diagnoses of subjects adjusted odds ratio.

^†^≥85 cm (male), ≥90 cm (female).
